# In Vivo Psychopharmacological Investigation of *Delphinium Denudatum* and *Amaranthus Spinosus* Extracts on Wistar Rats

**DOI:** 10.29252/NIRP.BCN.8.6.503

**Published:** 2017

**Authors:** Mohammad Abid, Ashok Kumar Gosh, Najam Ali Khan

**Affiliations:** 1. Department of Pharmacology and Clinical Research, School of Pharmaceutical Sciences, IFTM University, Moradabad, India.; 2. Department of Phytochemistry, School of Pharmaceutical Sciences, IFTM University, Moradabad, India.

**Keywords:** Antianxiety activity, Delphinium denudatum, Amaranthus spinosus, Flavonoids, Alkaloids

## Abstract

**Introduction:**

In our modern era, life style of human being changes and updates every day that may affect their health status. There is an incredible expectation that natural origin drugs lack undesirable effects not like synthetic drugs, though have the same potency and efficacy. No scientific data is available about the antianxiety properties of *Delphinium denudatum* root and *Amaranthus spinosus* leaves. In this regard, the present study was designed to carry out comparative and combined study on antianxiety properties of *Delphinium denudatum* root and *Amaranthus spinosus* leaves.

**Methods:**

*Delphinium denudatum* root and *Amaranthus spinosus* leaves were defatted with petroleum ether, and then extracted with hydroalcoholic solvent by soxhlation process. The hydroalcoholic extract of both drugs singly and in combination were evaluated for their anxiolytic effects on Wistar albino rats in doses of 200 and 400 mg/kg using different anti-anxiety tests like Elevated Plus Maze, Staircase, Actophotometer, and Light and Dark tests.

**Results:**

Both hydroalcoholic extracts possessed flavonoids, alkaloids, saponins, tannins, carbohydrates, proteins, amino acids, carbohydrates, steroids, sterols, etc. In the dose dependent manner, both the hydroalcoholic extracts produced good anxiolytic activity. The best result was obtained by a combination of them in higher dose.

**Conclusion:**

Hydroalcoholic extracts of *Delphinium denudatum* root and *Amaranthus spinosus* leaves and their combination may act as a potent anxiolytic agents in rats. *Amaranthus spinosus* was found to be more effective than *Delphinium denudatum*.

## 1. Introduction

In this modern era, life style of human being changes and updates every day that may affect their health status. Furthermore, the workload and accompanied problems can cause a variety of mental disorders. Anxiety has been conceptualized as a prevalent and serious disorder affecting the world population, independent of ethnicity. It is being considered as a cardinal symptom of many psychiatric disorders (Cassano, Pini, Saettoni, & Dell’Osso, 1999). There are plenty of drugs like alprax, clonazepam, benzodiazepines, and so on in the market for treating mental disorders such as depression, anxiety, insomnia, and stress. However, these drugs have a number of adverse effects like amnesia; dependence; tolerance; disturbance in respiratory, immune, and digestive systems; and so on (Dhawan, Dhawan, & Chhabra, 2003). Many people believe that natural origin drugs may lack any undesirable effect as compared to the synthetic drugs, though have the same potency and efficacy.

In Unani System of Medicine, Jadwar is considered as antipyretic, antiseptic, detergent, diuretic, exhilarant, solvent, anti-inflammatory, demulcent, sedative, analgesic, aphrodisiac, antidote, cardiotonic, and tonic for the brain. Traditionally, Jadwar is used for the treatment of mania, migraine, paralysis, epilepsy, pain, and as an antidote for snake bite and scorpion sting (Rauf, 2013). Jadwar belongs to the family *Ranunculaceae*, used in a number of Indian systems of medicines for different complaints and diseases (Qudsia & Jafri, 2006). Also, its root is used in various medical formulations in Unani and Ayurveda to reduce the withdrawal symptoms in people on de-addiction therapy (Zafar, Ahmad, & Siddiqui, 2003). Herbalists recommend the roots in the treatment of fungal infections, dysuria, calculi, asthma, cough, jaundice, and nervous problems (Rahman, Khan, & Kumar, 2007). *Khameera Gaozaban Ambari Jadwar Ood Saleeb Wala, Habb-e Jadwar, Habb-e Jawahar and Jawahar Mohra, Marham-e Jadwar, Zimad-e Warm-e Lozatain* are some of CNS acting formulations of Jadwar in Unani System of Medicine (Nadkarni, 1976).

The plant has many bioactive constituents, some of which are flavonoids, triterpenoids, alkaloids, including delphocurarine, staphisagrine, delphine, condelphine, denudatin and a diterpenoid alkaloid identical to condelphine (Singh & Chopra, 1962). Jadwar is reported to have some pharmacological activities like antibacterial, antifungal, anticonvulsive, hepatoprotective, as well as de-addiction of morphine, and so on (Rauf, 2013).

*Amaranthus spinosus* is an erect plant, found in different colors in various regions of Asia such as Japan, Indonesia, and India. In India, it is reported that the plant possesses a number of medicinal properties such as laxative, diuretics, antipyretics, antibronchitis, anticonvulsive, antidepressive and wound healing (Mishra, Verma, Mukerjee, & Vijayakumar, 2012; Mitra, 2013; Kumar, Lakshman, Velmurugan, Sridhar, & Gopisetty, 2014). It was reported to have hepatoprotective, antioxidant, antigenic and allergenic, antidiabetic, hyperlipidemic, spermatogenic, anti-inflammatory, anthelmintic, anti-malarial, heamatologic, analgesic, and antifertility effects (Kawade, Ghiware, Sarje, & Vadvalkar, 2013). In the traditional system of medicine, herbal formulation and the combined extracts of plants (rather than single drug) are used as drug of choice. However, there is no scientific data about antianxiety effect of these two plants. In this regard, the present study was designed to carry out comparative and combined study of *Delphinium denudatum* root and *Amaranthus spinosus* leaves for their antianxiety effect.

## 2. Methods

### 2.1. Preparation of extracts

The root of *Delphinium denudatum* was collected from local market of Moradabad, Uttar Pradesh and leaves of *Amaranthus spinosus* from IFTM University Botanical Garden. The parts of plants used were recognized and legitimated by the Botanist, Dr. Beena Kumari, Hindu College, and Dr. Ashok Kumar from IFTM University, Moradabad Uttar Pradesh. The botanical nomenclature of the plants, *Delphinium denudatum* and *Amaranthus spinosus*, were suitably recognized by matching with herbarium records and standard flora. The parts of the plants were dried under the shade, powdered, and passed through a 20-mesh sieve, then defatted with petroleum ether (60°C–80°C). Afterwards, defatted mark was extracted with hydroalcoholic solvent (Ethanol 95%, v/v: water, 1:1) in Soxhlet extractor. The extracts were filtered and concentrated separately by removing the solvents up to the dryness using a rotatory vacuum evaporator.

### 2.2. Phytochemical study

The hydroethanolic extracts were undergone phytochemical study using standard methods (Kokate, 1986; Evans, 2009).

#### 2.2.1 Test for flavonoids

##### 2.2.1.1 Shinoda Test

A few magnesium turnings were added into the to the test solution and then dropwise concentrated HCL was added until crimson, red, pink scarlet or occasionally green to blue color appeared after a few minutes.

##### 2.2.1.2 Alkaline Reagent Test

A few drops of NaOH (sodium hydroxide) was added to the test solution; strong yellow color was produced which disappeared after addition of a few drops of dilute acid indicating the presence of flavonoids.

#### 2.2.2 Test for saponins

##### 2.2.2.1 Foam Test

A little amount of extract was mixed with 20 mL of distilled water and shaken in a graduated cylinder for 15 min lengthwise. Formation of foam indicates the presence of saponins.

#### 2.2.3 Test for alkaloids

##### 2.2.3.1 Dragendorff’s Test

One milliliter of Dragendorff’s reagent is added to 1 mL of test solution; the appearance of an orange-red precipitate indicates the presence of alkaloids.

##### 2.2.3.2 Mayer’s Test

One milliliter of Mayer’s reagent (potassium mercuric iodide solution) was mixed with 1 mL of the test solution. Cream colored or whitish yellow precipitate indicates the presence of alkaloids.

#### 2.2.4 Test for steroids and sterols

##### 2.2.4.1 Salkowski Test

The extract in the chloroform was mixed and added the same volume of concentrated sulfuric acid; formation of bluish red to cherry color in chloroform layer and green fluorescent in the acid layer indicates the presence of steroids.

##### 2.2.4.2 Liebermann-Burchard Test

A few drops of chloroform was added to 1 g of test solution, then shaken well and added 3 mL of acetic anhydride, 3 mL of glacial acetic acid, heated and cooled under the tap water and put drops of concentrated sulfuric acid along the side of the test tube. Formation of bluish-green color shows the presence of sterol.

#### 2.2.5 Test for amino acids

##### 2.2.5.1 Ninhydrin Test

Two drops of freshly prepared 0.2% Ninhydrin re-agent (0.1% solution in n-Butanol) was added to a small quantity of extracted solution and heated. Appearance of blue color indicates the presence of proteins, peptides or amino acids.

#### 2.2.6 Test for carbohydrates

##### 2.2.6.1 Molisch’s Test

Two milliliter of the test solution was taken and mixed with 1 mL of a-naphthol solution, then pour concentrated sulfuric acid along the side of the test tube. In between the two liquids, purple or reddish violet color indicates the occurrence of carbohydrates.

#### 2.2.7 Test for protein

##### 2.2.7.1 Biuret Test

One milliliter of the test solution was added in the blue color mixture of 1 mL of 40% sodium hydroxide solution and 2 drops of 1% CuSO_4_ solution. Development of pinkish or purple violet color shows the occurrence of proteins.

##### 2.2.7.2 Millon’s Test

Sulfuric acid was added to 1 mL of test solution, then Millon’s reagent was added and heated up to the boil. A yellow color precipitate is produced which indicates the presence of protein.

#### 2.2.8 Test for tannins

##### 2.2.8.1 Extract+5% Ferric Chloride solution

One milliliter of 5% ferric chloride solution was mixed with 1 mL of the test solution; development of dark blue color or greenish black color products indicates the presence of tannins.

##### 2.2.8.2 Extract+Lead Acetate solution

A little amount of test solution was added to the basic lead acetate solution; production of white precipitate shows the presence of tannins.

### 2.3. Animals

The recent experimental work was done on either sex of Wistar albino rats (Kumar & Bhat, 2012; Keshetti, Garige, & Vattikuti, 2016; Kumar, Chatterjee, Singh, Chauhan, & Rai, 2013) weighing 150–200g. Animals were procured from the animal house of IFTM University, Moradabad and maintained on a natural day/night cycle (12:12 h) at room temperature, with free access to standard food pellets and water ad libitum. Before exposure to experiment, the animals were acclimatized for at least ten days. The experiments were carried out between 10:00 AM till 5:00 PM. The study was approved by the Institute Animal Ethics Committee, Department of Pharmacology and Clinical Research, College of Pharmacy, IFTM University, Moradabad. All animal care and experimental protocols were in compliance with the National Institute of Health (NIH) guidelines for the care and use of the laboratory animals. The animals were divided into 9 groups and each group contained 6 animals.

### 2.4. Acute toxicity studies

The test drugs was administered in the range of the doses (50–2000 mg/kg, PO) of roots *Delphinium denudatum* Extract (DDE) and leaves *Amaranthus spinosus* extract (ASE) to the animals and continuously observed for one and a half hourly intervals for 4 hours, then for any gross behavioral changes up to the 72 hours followed by 14 days for any mortality (Organization for Economic Cooperation and Development, 1993).

### 2.5. Antianxiety activity

#### 2.5.1. Experimental design for antianxiety activity

Animal were treated with tests and standard drugs for 7 consecutive days (once a day). The test was performed on the seventh day after 60 min administration of test drugs per oral by bulb tipped gastric gavage needle (18 gauge) and 30 min standard drug diazepam 2 mg/kg IP Kumar et al., 2013; Anuradha, Srikumar, Shankaranarayana Rao, & Lakshmana, 2007; Kumar, Maheshwari, & Singh, 2009).

#### 2.5.2 The anxiety inducing process

Prior to testing the antianxiety effect of each drug, rats were undergone the restraint-induced anxiety. Restraint-anxiety and immobilization procedure are most common to induce stress-related behavior, also biochemical, and physiological changes in the animals (Kvetnansky & Mikulaj, 1970).

Taken a cylindrical or semi-cylindrical tube with ventilation holes, then the animal is kept for 120–180 min for restraint stress. After this, the animals show the signs of elevated levels of anxiety in the Elevated Plus Maze (EPM) and other tests of anxiety models (Padovan & Guimarães, 2000).

Hence, rats were located in Plexiglas restrainers for 2 h at room temperature daily for seven days after administration of tests and standard drugs. Daily stress was induced for 2 hours and the experiments were performed on the seventh day.

#### 2.5.3 Elevated Plus Maze Test

The Elevated Plus Maze (EPM) is made up of 4 arms forming a plus sign with 2 open arms (5×10 cm) and 2 closed arms (5×10×l5 cm). It was positioned 40 cm above the floor. The animal was placed at the center of the EPM, head facing towards the open arms. The time spent in open arm was recorded during 5 min periods. Arm entry was counted when the animal had placed all of its four paws on it. The procedure was conducted in a sound attenuated room (Nade, Yadav, & Kawale, 2008). Animals were divided into 9 groups, and each group contained 6 animals. Group I was the control group which received the vehicle (tween 80 [2% in distilled water] 5 mL/kg orally). Group II was Anxiety Control (AC) group, treated with vehicle. Group III and IV received *Delphinium denudatum* Extract (DDE) (200 and 400 mg/kg, PO). Group V and VI were treated with *Amaranthus spinosus* Extract (ASE) (200 and 400 mg/kg, PO). Group VII and VIII were treated with combination of both drugs, i.e. C_1_ and C_2_ (50 and 100 mg/kg, PO), and group IX was the standard group which received diazepam (2 mg/kg, IP).

#### 2.5.4 Stair Case Test

The apparatus made up of five steps (2.5 cm high, 10 cm wide and 7.5 cm deep). The internal height of the walls is constant along the whole length of the staircase. The animals were placed on the first step of the apparatus. The number of steps climbed and the number of rears was counted over a 3 m period. A step was considered to be climbed only when the animal places all four paws on the step. In order to simplify the observation, the number of steps descended were not taken into account. The apparatus was cleaned for each animal in order to eliminate any olfactory cues, which might modify the behavior of the next animal (Rakotonirina, Bum, N., Rakotonirina, & Bopelet, 2001). Treatment and grouping of animals are the same as in EPM.

#### 2.5.5 Light and Dark Test (LDT)

The animal was put individually in the center of the apparatus. The time spent in light and dark boxes for a period of 5 min was observed (Bodhankar, Ambavade, Mhetre, & Tate, 2006). Treatment and grouping of animals are the same as in EPM.

#### 2.5.6 Test for locomotor activity

The animal was kept in actophotometer for 5 min to measure the locomotor activity. The apparatus contained light beams, which were connected to a counter for recording of light beam interruptions as a locomotor score (Kulkarni, 1987). Treatment and grouping of animals are the same as in EPM Test.

#### 2.5.7. Statistical analysis

One-way ANOVA followed by Dunnett’s Test were conducted and the results were expressed as Mean±SEM and P<0.05 was considered significant.

## 3. Results

### 3.1. Preliminary phytochemical study

The hydroalcoholic extract of both *Delphinium denudatum* root and *Amaranthus spinosus* leaves extracts showed the presence of alkaloids, tannins, flavonoids, saponins, proteins sterols and steroids.

### 3.2. Acute toxicity study

Both *Delphinium denudatum* root and *Amaranthus spinosus* leaves extracts were studied for acute toxicity up to the doses of 2000 mg/kg. The extracts were not found to produce any mortality or toxicity in experimental animals. Therefore, for experimental studies, 200 and 400 mg/kg doses of both drug extracts were utilized.

### 3.3. Anti-anxiety activity

In this test, on the seventh day of intervention, animals were treated with *Delphinium denudatum* Extracts (DDE) (400 mg/kg), *Amaranthus spinosus* Extracts (ASE) 200 mg/kg and their combination. C_1_ less significantly produced the antianxiety effect. ASE (400 mg/kg) more significantly augmented the time spent in open arm, whereas standard drug diazepam (2 mg/kg) and combination C_2_ showed a highly significant effect compared to the anxiety control group (Table 1).

**Table 1.** The effects of DDE, ASE, combination of both drugs and diazepam in elevated plus-maze test

**Groups (n=6)**	**Dose (PO)**	**Time Spent in Open Arms in 5-min Period (in Seconds) After 7 Days**
Control (vehicle), mL 5		27.50±5.40
Anxiety control, mL	5	15.00±3.8
AC+DDE, mg/kg	200	22.00±3.48^ns^
AC+DDE, mg/kg	400	39.85±8.39^*^
AC+ASE, mg/kg	200	32.00±7.45^*^
AC+ASE, mg/kg	400	64.85±5.39^**^
AC+C_1_, mg/kg	Each 50	35.40±6.25^*^
AC+C_2_, mg/kg	Each 100	90.85±5.39^***^
AC+Diazepam	2 mg, IP	87.00±2.82^***^

All values are presented as Mean±SEM.

*: P<0.05;

**: P<0.01;

***: P<0.001 compared with AC group (ANOVA followed by Dunnett’s test); ns: not significant

**Figure 1.**
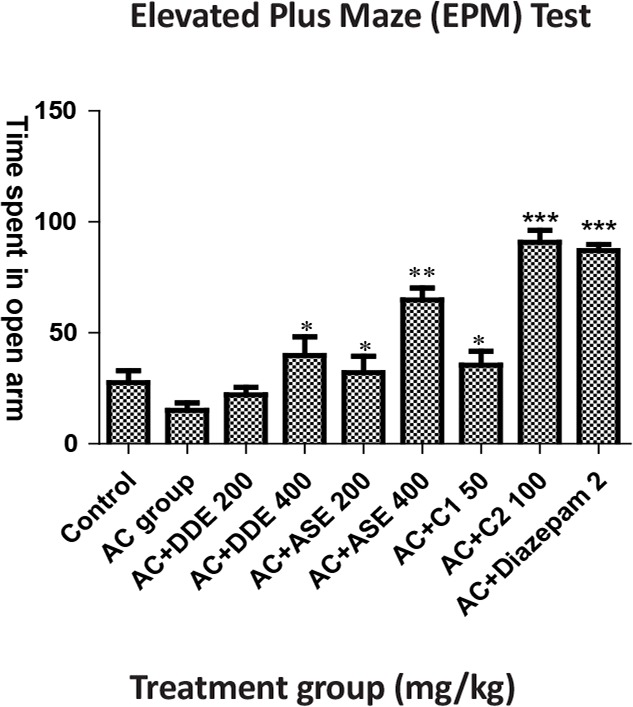
The effects of DDE, ASE, combination of both drug extracts and diazepam with respect to time spent in open arms of elevated plus maze test

**Figure 2.**
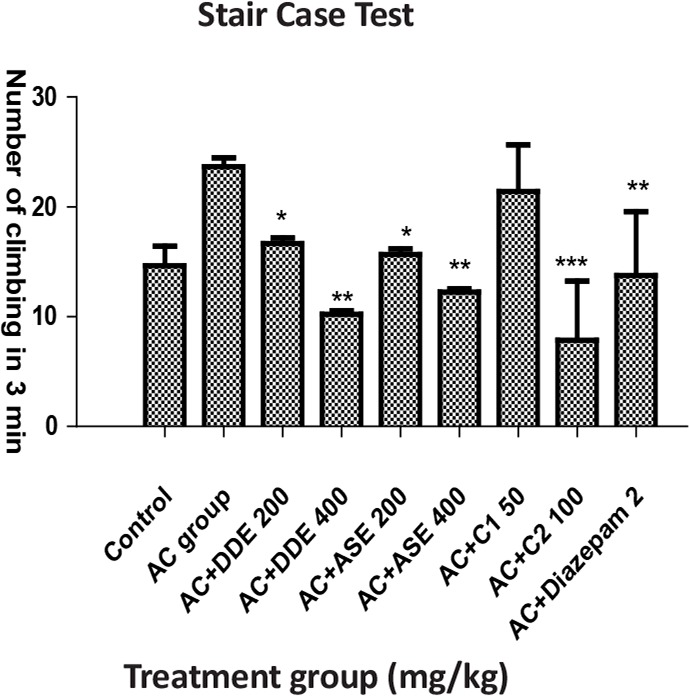
The effects of DDE, ASE, combination of both drugs with respect to the number of climbing in stair case test

In Stair Case Test (SCT), animals received DDE and ASE (200 mg/kg) that significantly decreased the number of climbing as well as rearing. DDE (400 mg/kg) significantly decreased the number of climbing and rearing. ASE 200 mg/kg did not produce significant effect, regarding the number of rearing, whereas diazepam (2 mg/kg) and C_2_ combination showed a highly significant effect. Combination C_1_ did not show a significant response compared to the Anxiety Control (AC) group (Table 2).

**Table 2.** The effects of DDE, ASE, and combination of both drugs as well as diazepam in results of stair case test

**Groups (n=6)**	**Dose (PO)**	**Number of Climbed Step in 3 min**	**Number of Rearing Step in 3 min**
Control (vehicle), mL	5	14.63±1.78	13.33±0.50
Anxiety control, mL	5	23.66±0.80	29.18±5.48
AC+DDE, mg/kg	200	16.66±0.50^*^	21.00±0.34^ns^
AC+DDE, mg/kg	400	10.23±0.30^**^	10.16±0.22^**^
AC+ASE, mg/kg	200	15.66±0.50^*^	16.34±7.74^*^
AC+ASE, mg/kg	400	12.23±0.30^**^	14±4.02^**^
AC+C_1_, mg/kg	Each 50	21.40±4.25^ns^	24.15±6.58 ns
AC+C_2_, mg/kg	Each 100	7.85±5.39^***^	6.11±4.30^***^
AC+Diazepam	2 mg, IP	13.76±5.80^**^	14.11±4.30^**^

All value are presented as the Mean±SEM.

*: P<0.05;

**: P<0.01;

***: P<0.001 compared with AC group (ANOVA followed by Dunnett’s Test)

**Figure 3.**
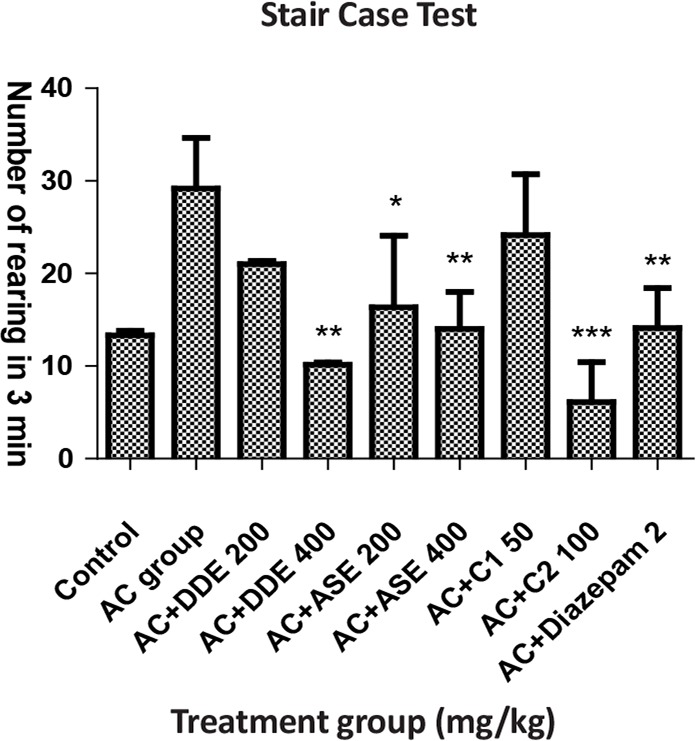
The effects of DDE, ASE, combination of both drugs and diazepam with respect to the number of rearing in stair case test

**Figure 4.**
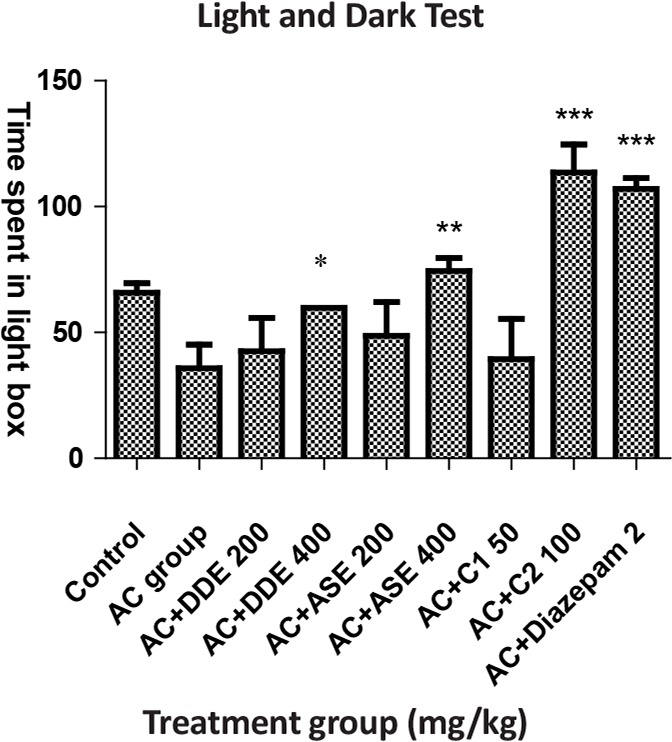
The effects of DDE, ASE, and combination of both drugs and diazepam with respect to the time spent in light compartment

In Light and Dark Test (LDT), animals were treated with DDE, ASE (200 mg/kg) and combination C_1_ did not show a significant effect. DDE (400 mg/kg) produced significant effect and ASE (400 mg/kg) more significant response, whereas diazepam (2 mg/kg) and combination C_2_ significantly increased time spent in the light compartment compared to the anxiety control (Table 3).

**Table 3.** The effects of DDE, ASE, combination of both drugs and diazepam in Light and Dark Test

**Groups (n=6)**	**Dose (PO)**	**The Time Spent in Light Box During 5-min Period (in Seconds) on the 7^th^ Day**
Control (vehicle), mL	5	65.80±3.80
Anxiety control, mL	5	35.80±9.40
AC+DDE, mg/kg	200	42.54±13.28^ns^
AC+DDE, mg/kg	400	59.85±8.39^*^
AC+ASE, mg/kg	200	48.70±13.42^ns^
AC+ASE, mg/kg	400	74.45±5.09^**^
AC+C_1_, mg/kg	Each 50	39.40±16.05 ^ns^
AC+C_2_, mg/kg	Each 100	113.55±11.09^***^
AC+Diazepam	2 mg, IP	107.07±4.22^***^

All values are presented as the Mean±SEM.

*: P<0.05;

**: P<0.01;

***: P<0.001 compared with AC group (ANOVA followed by Dunnett’s test)

In locomotor activity test, on the seventh day of intervention, animals treated with DDE and ASE (400 mg/kg) more significantly, and those with DDE and ASE (200 mg/kg) and combination C_1_ less significantly showed decreased locomotor activity, whereas those treated with diazepam (2 mg/kg) and combination C_2_ showed more significant decrease in locomotor activity compared to the anxiety control group indicating the sedative like effect. The combination C_2_ was found to be as effective as standard drug (Table 4).

**Table 4.** The effects of DDE, ASE, combination of both drugs and diazepam with respect to locomotor activity results

**Groups (n=6)**	**Dose (PO)**	**Locomotor Activity (s) Within 5 min**
Control (vehicle), mL	5	137.67±21.93
Anxiety control, mL	5	193.30±14.70
AC+DDE, mg/kg	200	124.50±8.44^*^
AC+DDE, mg/kg	400	96.00±8.77^**^
AC+ASE, mg/kg	200	117.50±7.24^*^
AC+ASE, mg/kg	400	81.00±8.77^**^
AC+C_1_, mg/kg	Each 50	112.50±4.04^*^
AC+C_2_, mg/kg	Each 100	76.00±8.07^***^
AC+Diazepam	2 mg (IP)	89.67±14.28^**^

All values are presented as the Mean±SEM.

*: P<0.05;

**: P<0.01;

***: P<0.001 compared with AC group (ANOVA followed by Dunnett’s test)

## 4. Discussion

The study showed that hydroalcoholic extracts of both drugs (DDE and ASE 200–400 mg/kg) and combination C_2_ (100 mg/kg) possessed anti-anxiety activity. High doses of both drugs (400 mg/kg) showed a more significant effect and their low doses (200 mg/kg) produced a less significant effect, and finally combination C_2_ (100 mg/kg, PO) and standard drug diazepam (2 mg/kg) showed a highly significant anxiolytic effect as compared to anxiety control group. The combination C_2_ was found to be more effective compared to the standard drug diazepam. Combination C_1_ produced some effect, but it was not significant compared to the anxiety control group.

EPM test is mainly used for evaluating psychomotor activity and emotional aspects of rodents (Nogueira and Vassilieff, 2000). When an animal spends more time in open arms it is assumed that it is in good mood and free from anxiety. In the EPM test, animals generally want to spend the time in the closed arms. This behavior of animals reflects an aversion towards the open arm that is generated by the fright of open places. The drugs that increase the time of spending in open arms are considered to be anxiolytic and if the drugs increased the time spent in closed arms, it reflects anxiogenic property (Nic Dhonnchadha, Bourin, & Hascoët, 2003). In this study, both drugs extracts (200 and 400 mg/kg) separately and in combination produced significant effect in a dose dependent manner compared to AC group.

SCT is used for evaluating anxiety-like behavior (number of rearing) and sedation (number of steps climbed). The increased number of rear related to the anxiety-like behavior and decreased number of climbed steps indicates higher calming effect/sedation (Gries, Condouris, Shey, & Houpt, 2005). The results of the recent work show that DDE, ASE, and combination of both drugs, i.e. C_2_, significantly decreased the number of rearing and number of steps climbed compared to AC group, i.e. these drugs may possess the anxiolytic and sedative properties.

Light and Dark Test method represents the natural habit of animals that like the dark place. During a 5-min period, animals are permitted to freely investigate a new atmosphere comprised two different compartments: protected (dark) and unprotected (light). Anxiolytic compounds change the natural habit of animals to light and increase the time spent in the light compartment (Barua et al., 2010). However, in this model, compared to anxiety control, only combination C_2_ and high doses of both drugs produced the significant effect, i.e. increase the time spent on the animals in the light box.

**Figure 5.**
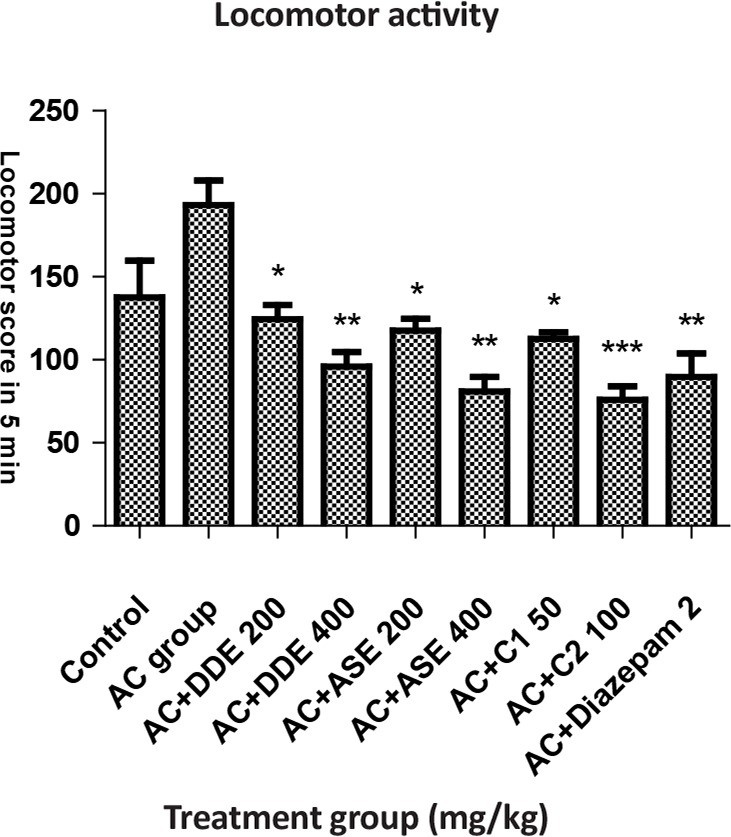
The effects of DDE, ASE, combination of both drugs and diazepam with respect to locomotor score in actophotometer test

The study of the spontaneous motor activity reflected that DDE and ASE (200 and 400 mg/kg) and combination of both drug extracts decreased the locomotor activity. The reduction of spontaneous motor activity could be related to the calmness/sedative effect of the DDE and ASE that characterize the lower anxiety-like actions (Rakotonirina et al., 2001). The antianxiety, antiepileptic, muscle relaxant, and sedative hypnotic effect of benzodiazepines drugs make them the most important GABA-A modulating drugs. Both DDE, ASE drugs and their combination C_2_ have similar effects like diazepam, hence the mechanism responsible for its anxiolytic activity of DDE and ASE (200 and 400 mg/kg) and combination of both drug extracts may be the same as benzodiazepines, mediated by GABA (inhibitory neurotransmitter) (Ramos et al., 2008). The results of the present work indicate that ASE is more effective compared to the DDE most probably because *Amaranthus spinosus*, contains different types of amino acids (Anonymous, 1988), flavonoids and phenolic compounds which have been reported to treat mental disorders (Priyanka, Sridhar, & Shankaraiah, 2012) and neurodegenerative diseases (Amresh, Reddy, Rao, & Singh, 2007).

These effects of both drugs could be due to the interaction of flavonoids, alkaloids, tannins, triterpenoids, and steroids (chemical constituents of the plants) with the GABA/benzodiazepine receptor complex in the brain (Kumar, 2000); however, flavonoids generally possessed the anxiolytic activity (Herrera et al., 2008; Mahendra & Bisht, 2011), hence both drugs contain flavonoids biochemical. Further studies are required to explain the exact mechanism of action of DDE and ASE (200 and 400 mg/kg) and their combination, also to isolate the main chemical constituents responsible for these types of activities.

The study showed that hydroalcoholic extracts of both drugs (DDE and ASE 200–400 mg/kg) and their combination C_2_ (100 mg/kg) possessed antianxiety effect. Further research is required to evaluate the real underlying mechanism of action of both drugs and responsible phytochemicals presents in the drugs. The synergistic effect of both drugs showed better effects. Also the results indicate that ASE is more effective than DDE.
